# Exploring the MRI-based vertebral bone quality score as a novel predictor of spongy cement distribution in osteoporotic vertebral compression fractures following percutaneous vertebral augmentation

**DOI:** 10.3389/fendo.2026.1926417

**Published:** 2026-09-07

**Authors:** Heng Wu, Wenjun Shen, Bin Hu, Shike Luo, Zhuolin Wang, Junwei Xiang, Yuquan Chen, Hua Yang, Chuanen Wang

**Affiliations:** 1 Department of Spine Surgery, The Affiliated Sports Hospital of Chengdu Sport University, Chengdu, China; 2 Department of Orthopedics, The Affiliated Hospital of Southwest Medical University, Luzhou, China; 3 School of Public Health and Preventive Medicine, Faculty of Medicine, Nursing & Health Sciences, Monash University, Melbourne, VIC, Australia; 4 Department of Quantitative Health Sciences, John A. Burns School of Medicine, University of Hawaii at Manoa, Honolulu, HI, United States

**Keywords:** bone cement distribution, osteoporotic vertebral compression fracture, percutaneous vertebral augmentation, predictor, vertebral bone quality score

## Abstract

**Background:**

Previous studies have demonstrated that a spongy cement distribution pattern is associated with superior clinical and radiological outcomes in patients with osteoporotic vertebral compression fractures (OVCFs). However, the relationship between MRI-based vertebral bone quality (VBQ) score and spongy cement distribution pattern remains unclear. This study aimed to evaluate the predictive value of the VBQ score for achieving a spongy cement distribution following percutaneous vertebral augmentation (PVA) and to identify independent influencing factors.

**Methods:**

We retrospectively reviewed patients with single-level OVCFs who underwent percutaneous vertebroplasty (PVP) or percutaneous kyphoplasty (PKP) from January 2020 to December 2024. Patients were divided into spongy distribution group (Spongy group, n=94) and non-spongy distribution group (Non-spongy group, n=142) based on postoperative anteroposterior and lateral X-rays. Potential factors, including age, sex, body mass index (BMI), bone mineral density (BMD), hypertension, diabetes, serum uric acid levels, smoking history, glucocorticoid use, fractured vertebral level, injected cement volume, surgical technique (PVP or PKP), VBQ score, intravertebral vacuum cleft (IVC), vertebral compression ratio (VCR), local cobb angle (LCA), and bone cement leakage, were collected and analyzed. Clinical outcomes, including visual analog scale (VAS) and Oswestry Disability Index (ODI) scores, were recorded and compared between groups at multiple postoperative time points. Complications such as adjacent vertebral fracture (AVF) and bone cement displacement (BCD) were recorded. Logistic regression analysis was performed to identify independent factors associated with the spongy cement distribution pattern. The predictive performance of VBQ score was evaluated by receiver operating characteristic (ROC) curve analysis.

**Results:**

A total of 236 patients were included. Univariate analysis revealed significant differences between the two groups in age, serum uric acid level, VBQ score, BMD T-score, cement volume, presence of IVC, VCR, LCA, and surgical technique (all *P* < 0.05). Binary logistic regression demonstrated that VBQ score (OR = 0.179, 95% CI 0.053–0.602, *P* = 0.005), VCR (OR = 0.770, 95% CI 0.677–0.875, *P* = 0.001), LCA (OR = 0.750, 95% CI 0.667–0.884, *P* = 0.001), IVC (OR = 0.171, 95% CI 0.045–0.648, *P* = 0.009), and cement volume (OR = 0.247, 95% CI 0.142–0.430, *P* = 0.001) were associated with a lower likelihood of achieving a spongy pattern, whereas higher BMD T-score (OR = 6.101, 95% CI 2.097-17.750, *P* = 0.001) and the PVP (OR = 4.914, 95% CI 1.374-17.574, *P* = 0.014) surgical technique were associated with a higher likelihood. The VBQ score demonstrated good predictive ability, with an area under the ROC curve (AUC) of 0.759 (95% CI: 0.697-0.821) and an optimal cut-off value of 3.155 (sensitivity: 72.3%, specificity: 73.2%). Patients with spongy cement distribution showed significantly better pain relief and functional improvement, as well as a lower incidence of complications (all *P* < 0.05).

**Conclusion:**

The preoperative VBQ score is a simple, non-invasive, and reliable predictor of spongy cement distribution pattern following PVA in patients with OVCFs. The VBQ score, LCA, VCR, IVC, and higher cement volume were associated with a lower likelihood of achieving a spongy pattern, whereas higher BMD T-score and the PVP technique were associated with a higher likelihood. These findings may assist surgeons in preoperative risk stratification and surgical planning.

## 1 Introduction

Osteoporotic vertebral compression fractures (OVCFs) are a type of fragility fracture associated with osteoporosis (OP), with over 8.9 million cases reported worldwide, imposing substantial socioeconomic burdens on the aging population (1–3). Clinically, OVCFs often result in severe back pain, progressive thoracolumbar kyphosis, paraspinal muscle atrophy, and a marked decline in quality of life (3–5). Conservative treatments, including bed rest, nonsteroidal anti-inflammatory drugs (NSAIDs), bracing, and physical therapy, often provide limited symptomatic relief and functional recovery (6–8). Since its introduction by Galibert et al. in 1987 (9), percutaneous vertebroplasty (PVP), along with percutaneous kyphoplasty (PKP), has become a widely adopted minimally invasive intervention for OVCFs, offering rapid pain relief through vertebral stabilization, height restoration, and kyphosis correction (10, 11). However, these procedures are not without complications, including cement leakage, vertebral height loss, residual kyphotic deformity, and adjacent vertebral fracture (AVF), which may adversely affect clinical outcomes (12, 13).

Growing evidence indicates that bone cement distribution patterns are critical determinants of postoperative outcomes following PVP and PKP. Lane et al. categorized cement distribution into spongy and blocky types based on postoperative radiographs (14). The spongy pattern is characterized by diffuse interdigitation of cement within the trabecular network, forming a porous and irregular structure, whereas the blocky pattern involves localized filling of a cavity, resulting in a dense and compact mass (15). Yu et al. demonstrated that the spongy pattern was associated with superior long-term radiological and clinical outcomes in the treatment of OVCFs (16). Similarly, Heo et al. reported in a 2-year follow-up study that the spongy distribution pattern was associated with greater restoration of vertebral body height and improved correction of local kyphosis compared with the blocky pattern (17). Consistently, Chen et al. found that the spongy group achieved better pain relief and functional recovery than the blocky group (18). Despite these findings, reliable and easily accessible preoperative predictors of cement distribution patterns remain lacking in clinical practice.

Recently, the vertebral bone quality (VBQ) score, derived from non-contrast T1-weighted lumbar magnetic resonance imaging (MRI), has emerged as a novel imaging marker for assessing bone quality (19). This metric is based on the principle that increased bone marrow fat infiltration is inversely correlated with bone strength and can be quantitatively evaluated on T1-weighted MRI (20). Kadri et al. further applied the VBQ score for osteoporosis screening and confirmed its reliability and effectiveness (21). The VBQ score has been validated in previous studies for osteoporosis screening and prediction of postoperative complications such as cage subsidence and pedicle screw loosening. Ehresman et al. demonstrated that patients with OVCF exhibited significantly higher VBQ scores compared with those without fractures, underscoring its potential utility in predicting OVCF risk (22). Moreover, accumulating evidence suggests that the VBQ score is also associated with postoperative outcomes, demonstrating predictive value for complications such as cage subsidence and pedicle screw loosening after spinal fusion (23).

However, the relationship between VBQ score and spongy bone cement distribution pattern following percutaneous vertebral augmentation (PVA) remains unclear. Therefore, this study aimed to evaluate the predictive value of the VBQ score for achieving a spongy cement distribution after PVA and to identify independent influencing factors.

## 2 Materials and methods

### 2.1 Study subjects

**Figure 1:**
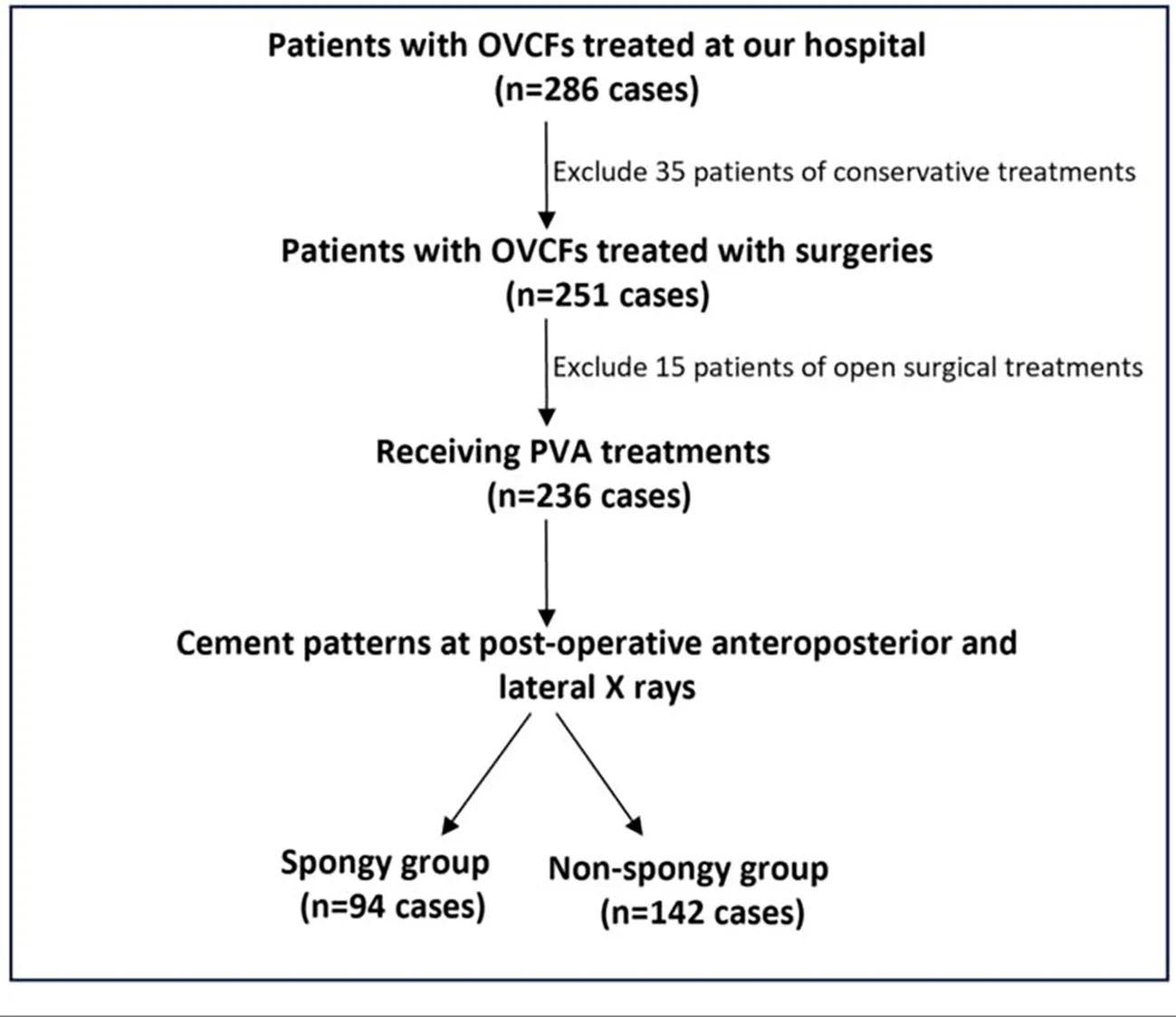
Flow diagram of patient election process.

**Figure 2:**
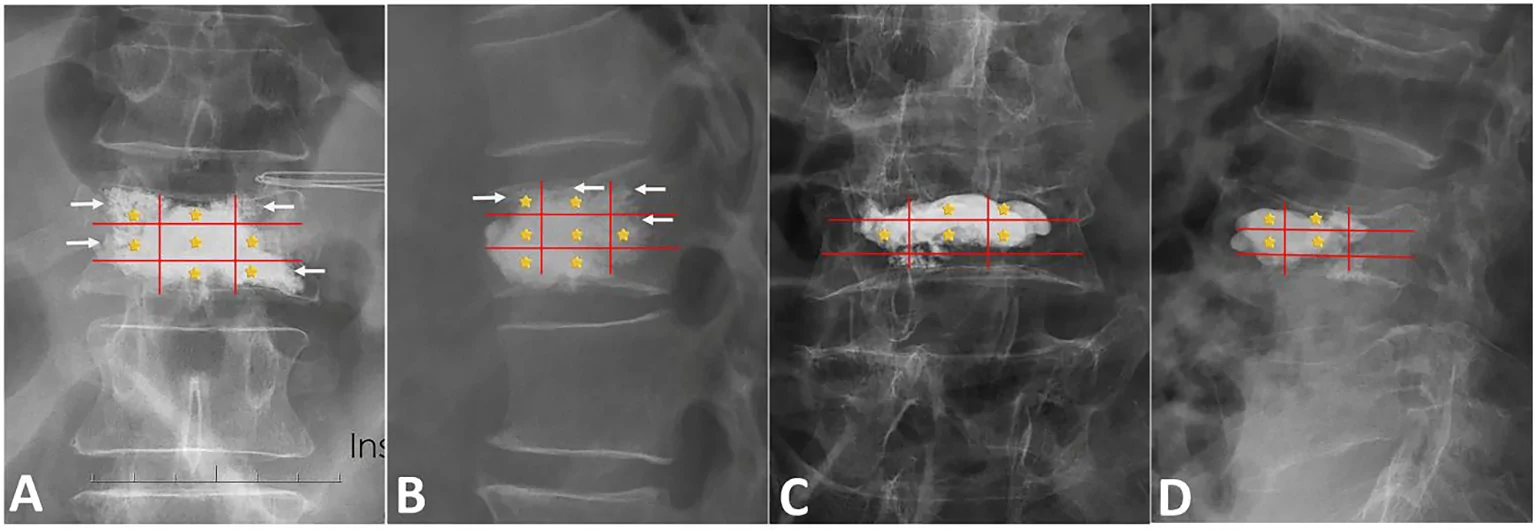
Spongy cement distribution method. The vertebral body was divided into 9 equal regions on post-operative anteroposterior and lateral X-rays. A region with **>** 50% cement filling was defined as an “effective” square (★). Dispersion into ≥ 6 effective squares was considered good. **(A, B)** Spongy pattern: bone cement interacted with cancellous bone (white arrow) and filled ≥ 6 effective squares. **(C, D)** Non-spongy pattern: bone cement did not interact with cancellous bone and filled < 6 effective squares.

This single-center retrospective study was approved by the Ethics Committee of the Affiliated Sports Hospital of Chengdu Sport University (NO. 202609). All procedures were conducted in accordance with the Declaration of Helsinki and relevant institutional guidelines. Written informed consent was obtained from all participants. *A* priori power analysis was performed using G*Power software (version 3.1.9.7) to estimate the required sample size. For the primary predictor, the VBQ score, a binary logistic regression model was assumed. With an anticipated odds ratio of 1.5 (representing a moderate effect size), a significance level (α) of 0.05, and a statistical power of 0.80, the minimum required sample size was calculated to be 231 patients. Between January 2020 and December 2024, a total of 286 patients with OVCFs were screened according to the inclusion and exclusion criteria. After excluding 35 patients who received conservative management and 15 patients who underwent open surgical procedures, 236 patients who were treated with PVA were finally enrolled, satisfying the estimated sample size. Based on the distribution patterns of bone cement on postoperative anteroposterior and lateral X-rays, the cohort was divided into two groups: the “Spongy group” (N = 94) and the “Non-spongy” group (N = 142) (**Figure 1**) (18). The vertebral body was divided into nine equal regions on postoperative anteroposterior and lateral X-rays (24). A region with >50% cement filling was defined as an “effective” square, with the percentage of cement filling in each region estimated visually by two independent observers. The nine regions were assessed independently on both anteroposterior and lateral views, and the final classification was based on a combined assessment: only patients with ≥6 effective squares on both views were classified as having a spongy pattern. The spongy pattern was defined as bone cement interacting with cancellous bone and filling ≥6 effective squares (**Figures 2A, B**). In contrast, the non-spongy pattern was characterized by bone cement not interacting with cancellous bone and filling <6 effective squares (**Figures 2C, D**). Inter-observer reliability for the cement distribution classification (spongy vs. non-spongy) was assessed using Cohen’s kappa coefficient in a randomly selected subset of 50 patients. The Kappa value was 0.836 (95% CI: 0.751–0.921), indicating excellent inter-observer agreement.

The inclusion criteria were as follows: (1) Preoperative thoracic or lumbar pain without neurological compression symptoms. (2) Bone mineral density (BMD) T-score of < −2.5. (3) Single-level vertebral fracture corresponding to the symptomatic segment. (4) MRI evidence of vertebral edema, including low signal intensity on T1-weighted images, low or mixed signal on T2-weighted images, and high signal on fat-suppressed sequences. (5) Concordance between the symptomatic vertebra identified on physical examination and imaging studies. (6) No history of prior vertebral fractures.

The exclusion criteria were as follows: (1) Patients with spinal metastases. (2) Multi-segmental vertebral fractures. (3) Patients with local or systemic infections. (4) Incomplete clinical data. (5) History of prior spinal surgery.

### 2.2 Surgical procedure

All surgeries were performed under local infiltration anesthesia via a unilateral transpedicular approach by the same senior surgeon. Patients were positioned prone, and standard aseptic preparation was performed. The target vertebra was identified using C-arm fluoroscopy in combination with preoperative radiographs. Under fluoroscopic guidance, the puncture needle was inserted at the 10 o’clock position of the left pedicle and the 2 o’clock position of the right pedicle, aiming for the anterior one-third of the affected vertebral body. Upon reaching the target area, a guide needle, followed by a dilator and a working cannula, were sequentially introduced under fluoroscopic guidance. For PKP, a balloon was then inserted to restore vertebral height, whereas no balloon was used in PVP. Bone cement was prepared to the wire-drawing phase and gradually injected into the vertebral body under continuous fluoroscopic monitoring. Patient lower limb sensation and motor function were observed in real time to ensure no spinal cord compression occurred. The procedure was immediately terminated if the cement approached within 1 mm of the posterior vertebral body wall or if cement leakage was detected, and the total volume of injected cement was recorded. Postoperatively, patients were instructed to remain in bed for 1 day, followed by a bone-strengthening regimen including calcium carbonate, vitamin D3 tablets, and zoledronic acid injections (25).

### 2.3 Observation indicators

The following patient data were collected: age, sex, body mass index (BMI), BMD, hypertension, diabetes, serum uric acid levels, smoking history, glucocorticoid use, fractured vertebral level, injected cement volume, surgical technique (PVP or PKP), VBQ score, intravertebral vacuum cleft (IVC), vertebral compression ratio (VCR), local cobb angle (LCA), and bone cement leakage, were collected and analyzed. Clinical outcomes, including visual analog scale (VAS) and Oswestry Disability Index (ODI) scores, were assessed at three standardized time points: preoperatively, 3 days postoperatively, and at the final follow-up (mean: 16.32 ± 3.73 months, range: 10–24 months). Complications including AVF and bone cement displacement (BCD) were recorded throughout the entire follow-up period.

**Figure 3:**
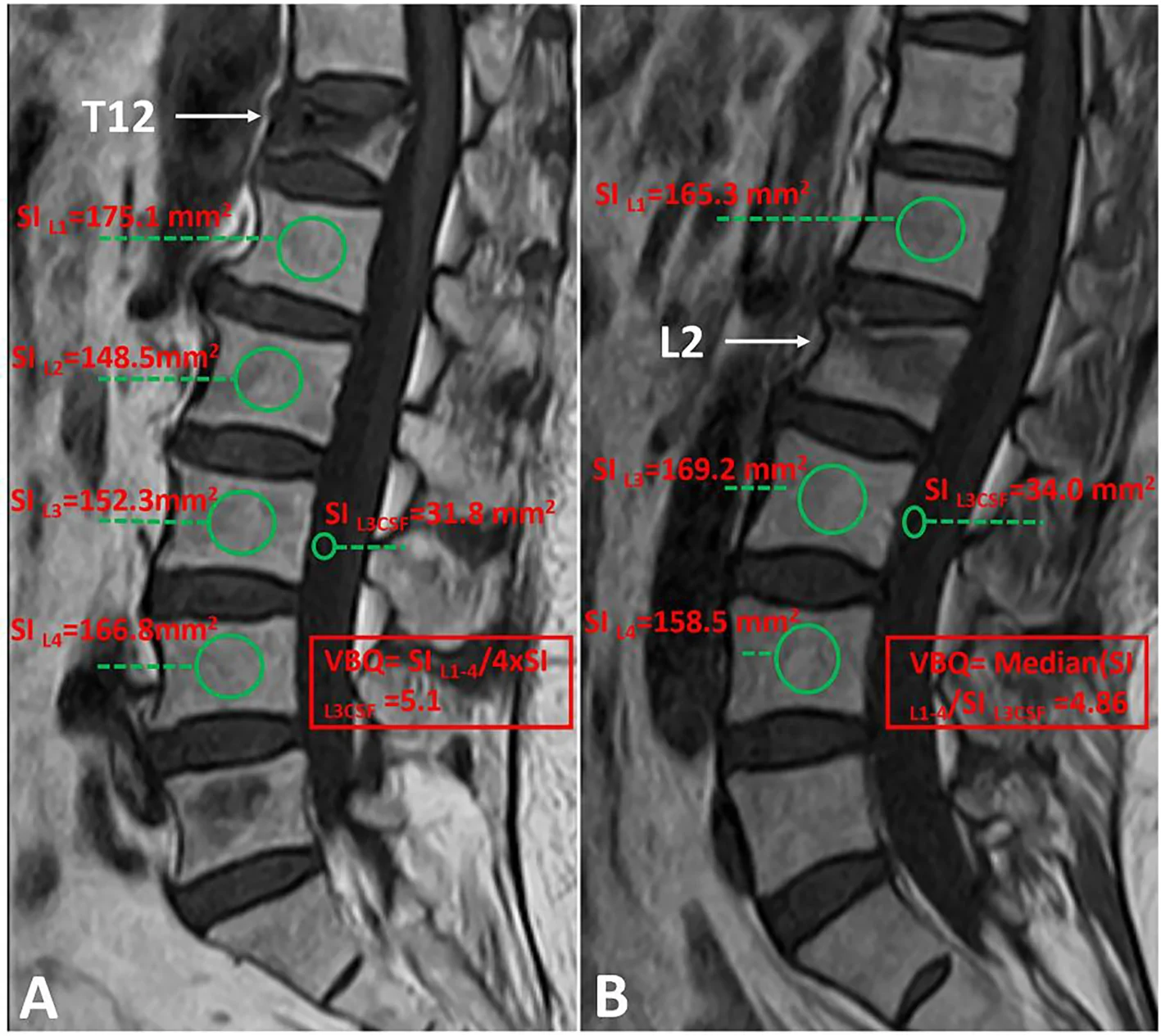
VBQ score measurement on non-contrast T1-weighted MRI. **(A)** For fractures outside the L1-L4 segment, the VBQ score was calculated as the mean SI of L1-L4 divided by the SI of CSF at L3. **(B)** For fractures within the L1-L4 segment, the VBQ score was determined by the median SI of L1-L4 divided by the SI of CSF at L3.

The VBQ score was calculated using non-contrast T1-weighted lumbar MRI (19). When the fractured vertebra was located outside the L1-L4 range, the average signal intensities (SI) within the regions of interest (ROI) in the medullary portions along the median sagittal plane of the vertebral bodies from L1 to L4 were measured. Simultaneously, the cerebrospinal fluid (CSF) SI was assessed at the L3 level. If nerve root compression or obstruction impeded accurate measurement of CSF signal intensity at the L3 level, the L2 or L4 levels were used instead. The VBQ score was then calculated by dividing the average SI from L1 to L4 by the CSF SI at the L3 level (26) (**Figure 3A**). If the fractured vertebra was located within the L1-L4 range, it was excluded from the calculation. In such cases, the median SI value from L1 to L4 was recorded and divided by the CSF SI at the L3 level (27) (**Figure 3B**). All MRI scans were performed with 3-mm slice thickness. For ROI placement, the observer identified the mid-sagittal slice of each vertebral body and manually traced an oval ROI within the cancellous bone region, carefully excluding the cortical bone, basivertebral veins, and any fracture lines. The ROI size was standardized to approximately 80–100 mm², with adjustments made according to vertebral body dimensions. For CSF signal measurement, the ROI was placed in the central portion of the spinal canal at the L3 level, carefully avoiding nerve roots, the dural sac margin, and any degenerative structures. In cases of severe spinal canal stenosis or osteoporotic degeneration causing inhomogeneous CSF signal, the measurement was performed at the L2 or L4 level as an alternative, and the mean of repeated measurements was recorded to minimize variability. Two physicians independently assessed the VBQ score, both blinded to patient information. The mean value of their assessments was subsequently recorded. Inter-observer reliability for the VBQ score measurements was assessed using the intraclass correlation coefficient (ICC) based on a two-way mixed-effects model for consistency. The ICC for single measures was 0.835 (95% CI: 0.726–0.903), indicating good inter-observer reliability.

**Figure 4:**
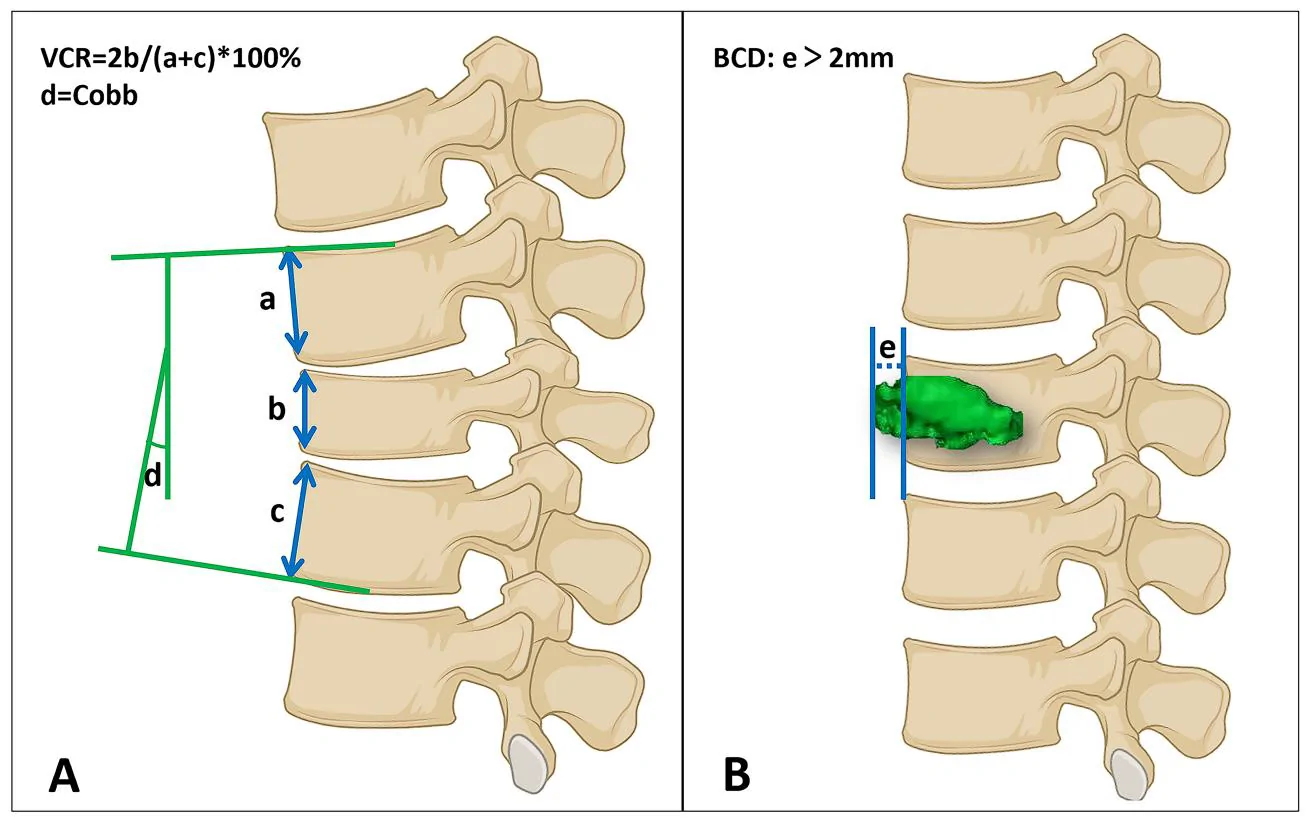
Radiographic measurements of vertebral compression ratio (VCR), Local cobb angle (LCA), and bone cement displacement (BCD). **(A)** VCR was calculated as twice the anterior height of the fractured vertebra (b) divided by the sum of the anterior heights of the adjacent upper (a) and lower (c) vertebrae: VCR = 2a/(b + c)×100%. The LCA was measured as shown (d). **(B)** BCD was defined as bone cement displacement >2 mm beyond the anterior vertebral wall (e).

The method of radiographic measurements of VCR, LCA, and BCD were performed as follows. The VCR was calculated as twice the anterior height of the fractured vertebra (b) divided by the sum of the anterior heights of the adjacent upper (a) and lower (c) vertebrae, expressed as VCR = 2a/(b + c)×100% (**Figure 4A**). The LCA was defined as the angle between the superior endplate of the vertebra above the fractured vertebra and the inferior endplate of the vertebra below the fractured vertebra (**Figure 4A**). BCD was considered significant when the anterior edge of the bone cement moved more than 2 mm from the anterior edge of the vertebral body, as indicated in prior studies (28) (**Figure 4B**).

### 2.4 Statistical analysis

All data were analyzed using IBM SPSS Statistics 25.0 and GraphPad Prism version 9.5. Normally distributed continuous variables are presented as mean ± standard deviation, while categorical variables are expressed as frequencies or proportions. Data normality was assessed with the Shapiro–Wilk test. Group comparisons were performed using the independent samples t-test for normally distributed continuous variables and the Mann–Whitney U test for non-normally distributed continuous variables. Categorical variables were compared using the chi-square test. Variables that showed statistically significant differences in univariate analyses were subsequently entered into a binary logistic regression model to identify independent predictors for spongy distribution pattern. The discrimination ability was evaluated using the area under the curve (AUC) of the receiver operating characteristic (ROC). Model calibration was assessed using the Hosmer-Lemeshow goodness-of-fit test, and the Nagelkerke R² was calculated to estimate the proportion of variance explained by the model. The sample size was determined *a* priori based on the primary predictor (VBQ score) with an anticipated odds ratio of 1.5 (moderate effect size), as detailed in the Study Subjects section. Youden’s index was used to determine the optimal cut-off values from the ROC curves. Statistical significance was set at *P* < 0.05.

## 3 Results

### 3.1 Univariate analysis

A total of 236patients with complete data were enrolled in this study: 94 patients in the Spongy group and 142 patients in the Non-spongy group. All patients underwent successful surgery and were followed up without any major complications. The overall mean follow-up duration was 16.32 ± 3.73 months (range: 10–24 months), with no significant difference between the Spongy group and the Non-spongy group (16.80 ± 3.80 months, range: 10–24 months, vs. 16.00 ± 3.80 months, range: 10–24 months, *P*>0.05). The clinical and radiographic outcomes are summarized in **Table 1**. No significant differences were observed between the two groups regarding sex, BMI, hypertension, smoking history, glucocorticoid use, fracture level, and bone cement leakage (all *P* > 0.05). However, age, serum uric acid level, VBQ score, BMD T-score, cement volume, presence of IVC, vertebral VCR, LCA, and surgical technique were significantly associated with the spongy distribution pattern (all *P* < 0.05).

**Table 1:** Univariate analysis of spongy bone cement distribution pattern after PVA for OVCFs.

Variables	Spongy group (n=94)	Non-spongy group (n=142)	χ²/t value	*P* value
Age (years)	69.80 ± 7.30	73.90 ± 7.00	-4.330	**0.001**
Sex			0.292	0.589
Male [n (%)]	35 (37.23%)	48 (33.80%)		
Female [n (%)]	59 (62.77%)	94 (66.20%)		
BMI (kg/m^2^)	23.98 ± 2.63	23.80 ± 2.90	0.475	0.635
BMD T-score	- (2.80 ± 0.50)	- (3.30 ± 0.60)	6.686	**0.001**
Hypertension [n (%)]	49 (52.13%)	83 (58.45%)	0.917	0.338
Diabetes [n (%)]	54 (57.45%)	62 (43.66%)	4.300	**0.038**
Serum uric acid (umol/L)	350.20 ± 62.30	324.50 ± 65.80	3.000	**0.003**
Smoking history [n (%)]	25 (26.60%)	44 (30.99%)	0.527	0.468
Glucocorticoid use [n (%)]	8 (8.51%)	18 (12.68%)	1.001	0.317
Fractured level			0.243	0.622
T11-L2 [n (%)]	38 (40.43%)	62 (43.66%)		
Other [n (%)]	56 (59.57%)	80 (56.34%)		
Cement volume (mL)	3.50 ± 1.20	4.90 ± 1.10	-9.224	**0.001**
PVP [n (%)]	67 (71.28%)	82 (57.75%)	4.449	**0.035**
VBQ score	2.95 ± 0.50	3.45 ± 0.55	-7.088	**0.001**
IVC [n (%)]	24 (25.53%)	54 (38.02%)	3.992	**0.046**
VCR (%)	33.03 ± 4.71	38.73 ± 5.43	-8.316	**0.001**
LCA (°)	16.80 ± 5.00	23.90 ± 6.90	-8.592	**0.001**
Bone cement leakage [n (%)]	14 (14.89%)	28 (19.72%)	0.900	0.343

VBQ, vertebral bone quality score; BMD, bone mineral density; VCR, vertebral compression ratio; LCA, local cobb angle; IVC, intravertebral vacuum cleft; PVP, percutaneous vertebroplasty. Bold values indicate *P* < 0.05, considered statistically significant.

### 3.2 Binary logistic regression analysis

**Figure 5:**
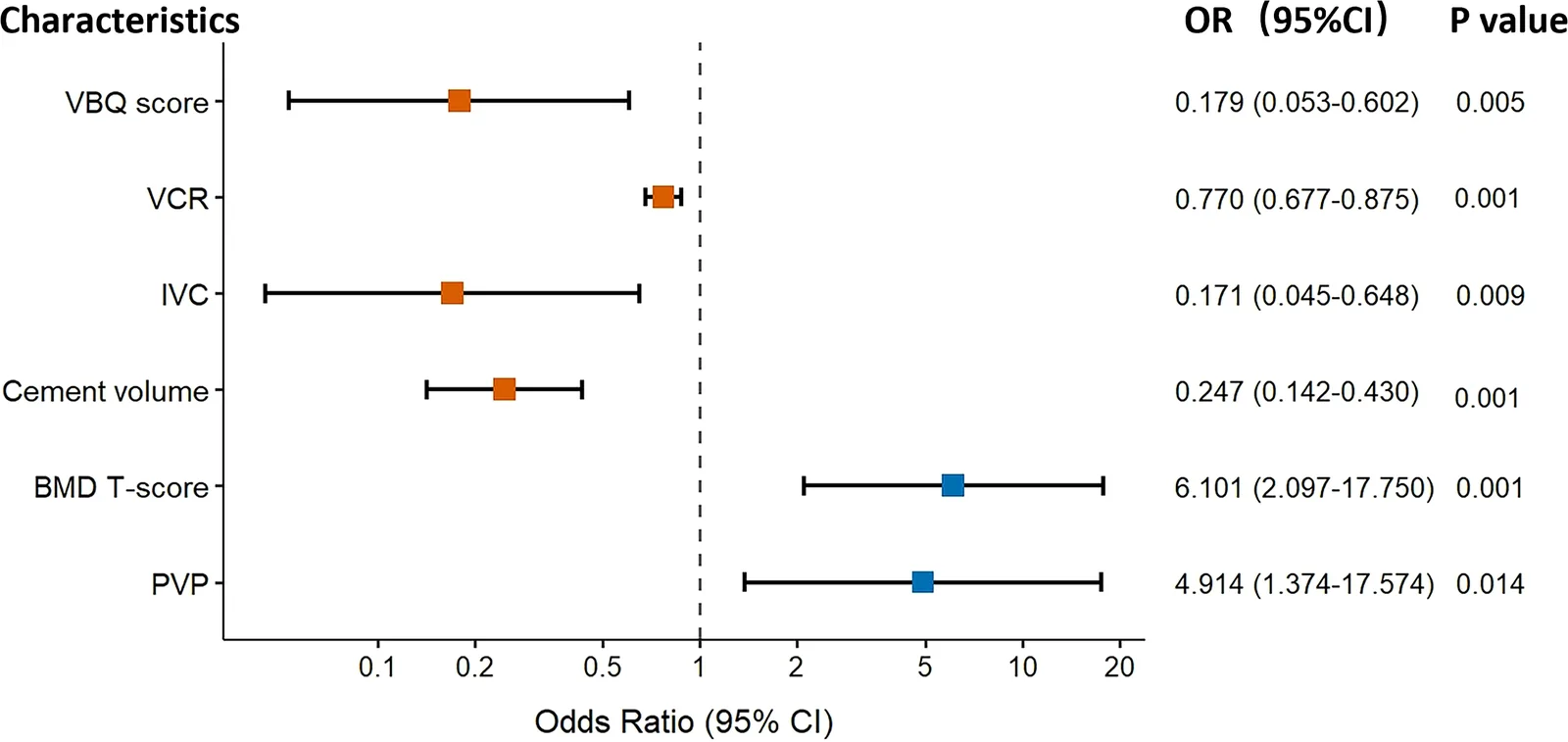
Forest plot for the independent predictors of spongy cement distribution.

All variables with *P* < 0.05 in univariate analysis were entered into the initial regression model. Although diabetes showed a significant association in univariate analysis (*P* = 0.038), it was not retained as an independent predictor in the final model (OR = 1.159, 95% CI: 0.372–3.611, *P* = 0.799). The independent predictors of spongy cement distribution following PVA for OVCFs are presented in **Table 2** and **Figure 5**. The following variables were identified as were associated with a lower likelihood of achieving a spongy pattern: VBQ score (OR = 0.179, 95% CI 0.053–0.602, *P* = 0.005), VCR (OR = 0.770, 95% CI 0.677–0.875, *P* = 0.001), LCA (OR = 0.750, 95% CI 0.667–0.884, *P* = 0.001), IVC (OR = 0.171, 95% CI 0.045–0.648, *P* = 0.009), and cement volume (OR = 0.247, 95% CI 0.142–0.430, *P* = 0.001). In contrast, higher BMD T-score (OR = 6.101, 95% CI 2.097–17.750, *P* = 0.001) and the PVP (OR = 4.914, 95% CI 1.374–17.574, *P* = 0.014) surgical technique were associated with a higher likelihood of spongy cement distribution. The logistic regression model demonstrated good calibration, as indicated by the Hosmer-Lemeshow goodness-of-fit test (χ² = 8.141, df = 8, *P* = 0.420). The Nagelkerke R² was 0.836, indicating that the model explained 83.6% of the variance in cement distribution, suggesting excellent explanatory power.

**Table 2:** Binary logistic regression analysis of spongy bone cement distribution pattern after PVA for OVCFs.

Variables	B	SE	Wald value	OR (95% CI)	*P* value
Age	-0.079	0.044	3.178	0.924 (0.847-1.008)	0.075
Diabetes	0.147	0.580	0.065	1.159 (0.372-3.611)	0.799
BMD	1.808	0.619	11.017	6.101 (2.097-17.750)	**0.001**
Serum uric acid	0.006	0.004	1.808	1.006 (0.997-1.014)	0.179
VBQ	-1.720	0.619	7.735	0.179 (0.053-0.602)	**0.005**
IVC	-1.764	0.679	6.757	0.171 (0.045-0.648)	**0.009**
Cement volume	-1.396	0.282	24.538	0.247 (0.142-0.430)	**0.001**
VCR	-0.261	0.065	16.010	0.770 (0.677-0.875)	**0.001**
LCA	-0.287	0.060	22.940	0.750 (0.667-0.884)	**0.001**
PVP	1.592	0.650	5.995	4.914 (1.374-17.574)	**0.014**

VBQ, vertebral bone quality score; BMD, bone mineral density; VCR, vertebral compression ratio; LCA, local cobb angle; IVC, intravertebral vacuum cleft; PVP, percutaneous vertebroplasty. Nagelkerke R² = 0.836. Bold values indicate *P* < 0.05, considered statistically significant.

### 3.3 Roc curve and correlation between VBQ Score and BMD

**Figure 6:**
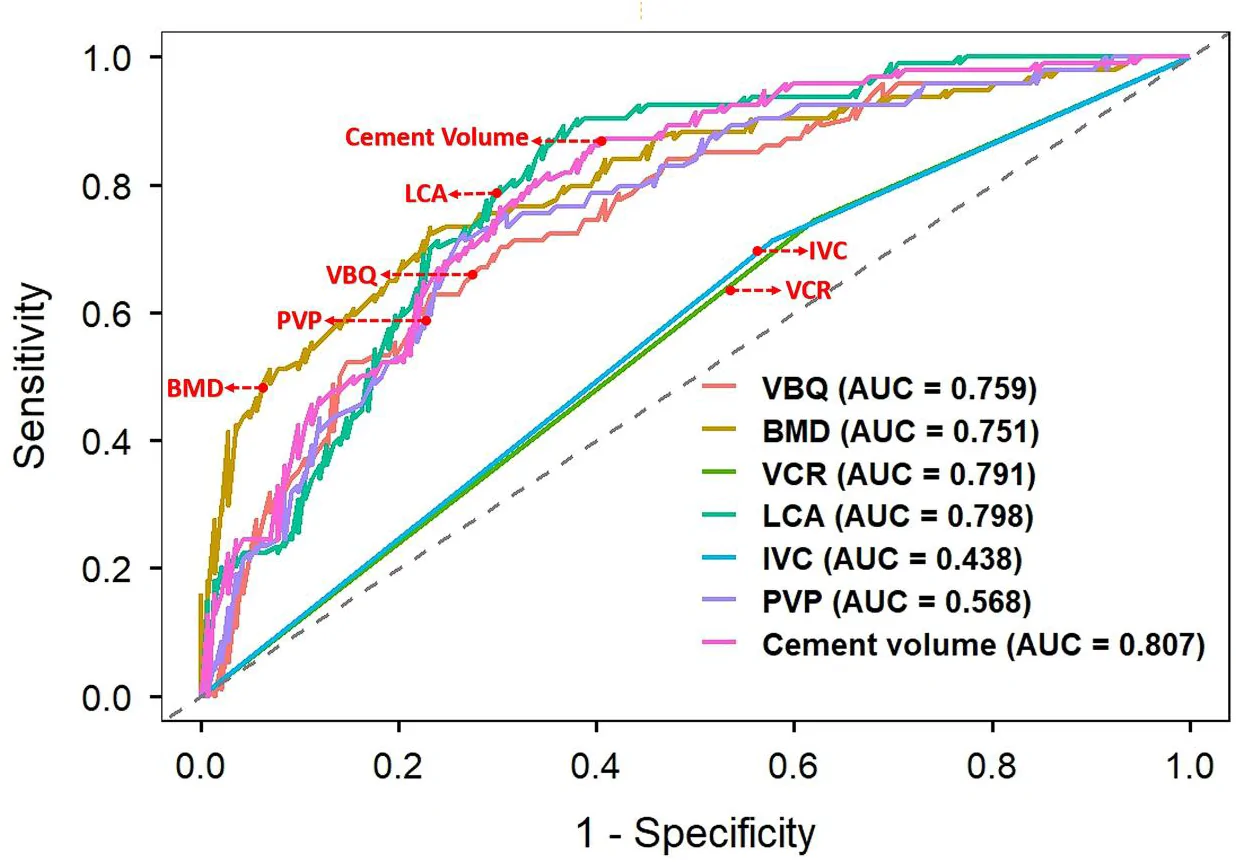
ROC curve for the independent predictors of spongy cement distribution.

The ROC curve was employed to assess the predictive performance of logistic regression for spongy cement distribution following PVA for OVCFs (**Figure 6**) (**Table 3**). The Youden’s index was calculated as “sensitivity – (1-specificity)” (29), and the optimal cutoff values were determined by identifying the point on the ROC curve corresponding to the highest Youden’s index. The ROC analysis showed that the AUC of the VBQ score was 0.759 (95% CI 0.697–0.821), with a sensitivity of 0.723, specificity of 0.732, and an optimal cutoff value of 3.155. The AUC of BMD was 0.751 (95% CI 0.688–0.814), sensitivity 0.702, specificity 0.679, and an optimal cutoff of −3.025. The AUC of VCR was 0.791 (95% CI 0.734–0.848), with sensitivity 0.872, specificity 0.599, and an optimal cutoff of 37.865%. The AUC of LCA was 0.798(95% CI 0.742–0.854), with sensitivity 0.894, specificity 0.634, and an optimal cutoff of 21.825°. The AUC of IVC was 0.438 (95% CI 0.363–0.512), with sensitivity 0.255 and specificity 0.620. The AUC of PVP was 0.568 (95% CI 0.494–0.642), with sensitivity 0.713 and specificity 0.577. The AUC of cement volume was 0.807 (95% CI 0.749–0.865), sensitivity 0.232, specificity 0.768, and an optimal cutoff of 4.095.

**Table 3:** ROC curve of spongy bone cement distribution pattern after PVA for OVCFs.

Variables	AUC	P value	95% CI	Sensitivity	Specificity	Optimal cutoff value	Youden’s index
Continuous variables							
VBQ	0.759	0.001	0.697-0.821	0.723	0.732	3.155	0.455
BMD	0.751	0.001	0.688-0.814	0.702	0.679	-3.025	0.399
VCR	0.791	0.001	0.734-0.848	0.872	0.599	37.865	0.471
LCA	0.798	0.001	0.742-0.854	0.894	0.634	21.825	0.528
Cement volume	0.807	0.001	0.749-0.865	0.232	0.768	4.095	0.502
Categorical variables							
IVC	0.438	0.038	0.363-0.512	0.255	0.620	N/A	N/A
PVP	0.568	0.079	0.494-0.642	0.713	0.577	N/A	N/A

VBQ, vertebral bone quality score; BMD, bone mineral density; VCR, vertebral compression ratio; LCA, local cobb angle. IVC, intravertebral vacuum cleft; PVP, percutaneous vertebroplasty.

**Figure 7:**
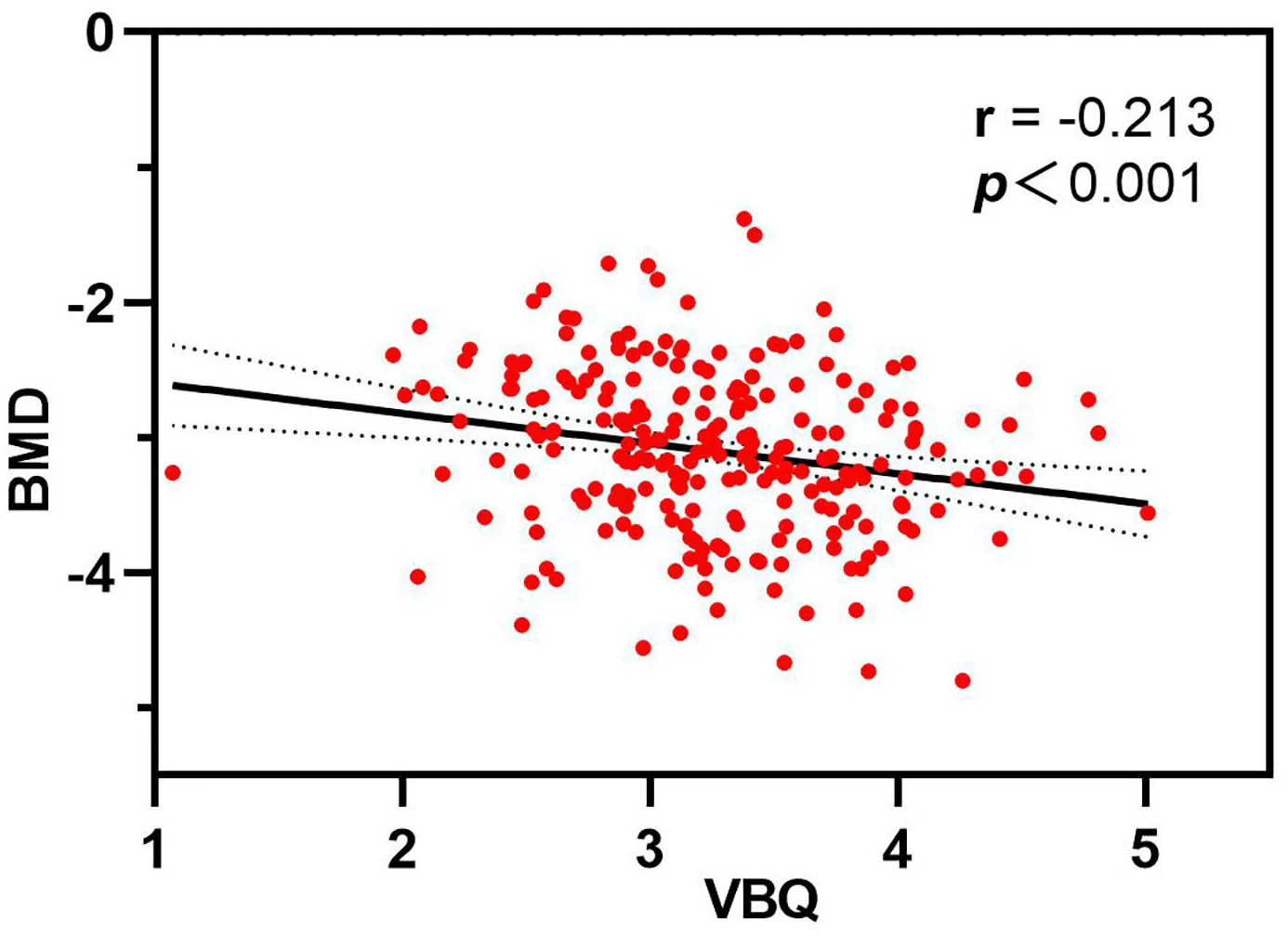
Scatter plot showing the correlations between VBQ score and BMD T score. The VBQ score is negatively correlated to BMD T score.

**Figure 7** illustrates the correlation between the VBQ score and BMD. A negative correlation was observed between the VBQ score and BMD (r=-0.213; *P* < 0.001).

### 3.4 Clinical outcomes between two groups

**Figure 8:**
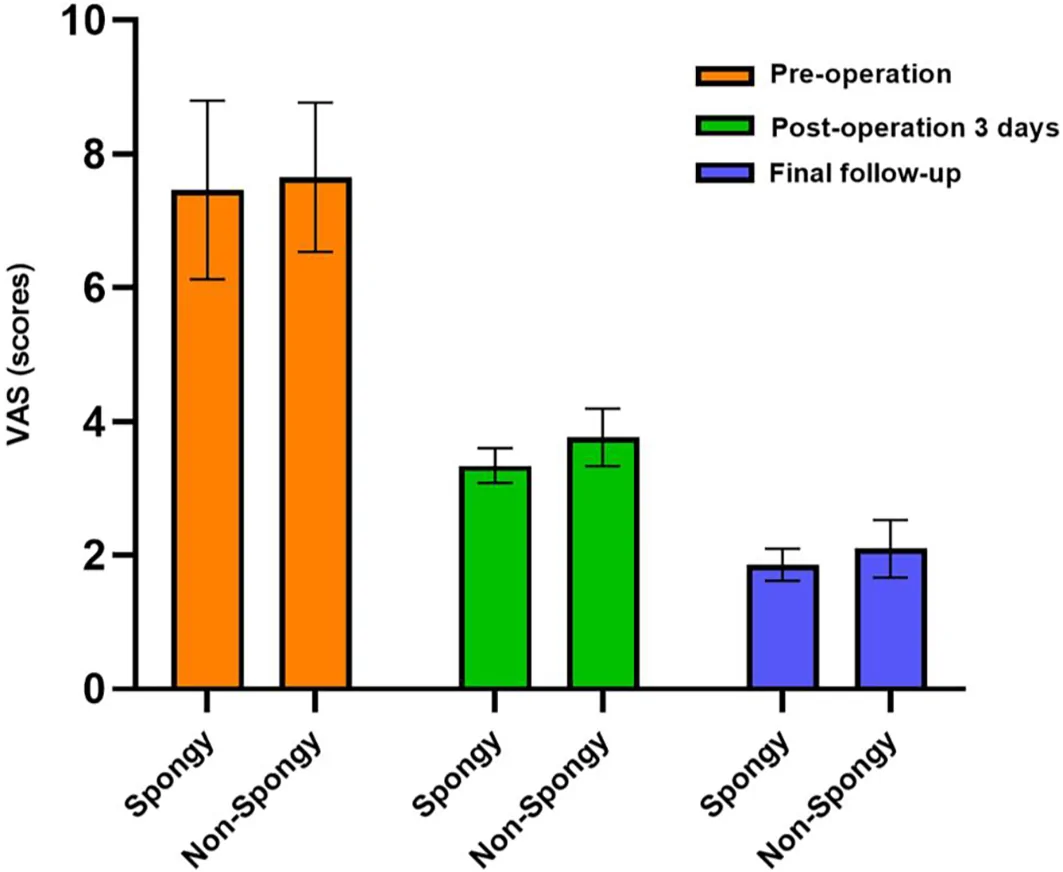
Histograms for VAS and ODI scores between spongy group and non-spongy group. Error bars represent standard deviations (SD).

The clinical outcomes of the two groups are summarized in **Table 4** and **Figure 8**. Both VAS and ODI scores showed no significant differences between the groups preoperatively (*P* > 0.05). The “Spongy” group demonstrated significant improvements in VAS and ODI scores at 3 days postoperatively the final follow-up (all *P* < 0.05). Additionally, the “Spongy” group had a significantly lower incidence of BCD compared with the “Non-spongy” group (6 cases, 6.38% vs. 21 cases, 14.80%; *P* < 0.05). No significant difference was observed in AVF incidence between the two groups (8 cases, 8.51% vs. 17 cases, 11.97%; *P* > 0.05).

**Table 4:** Clinical outcomes and complications between spongy group and non-spongy group.

Variables	Spongy group (n=94)	Non-spongy group (n=142)	χ²/t value	*P* value
VAS score (scores)			–	
Pre-operation	7.46 ± 1.34	7.65 ± 1.12	-1.176	0.241
Post-operation 3 days	3.34 ± 0.26	3.76 ± 0.43	8.516	**0.001**
Final follow-up	1.86 ± 0.24	2.10 ± 0.43	-4.927	**0.001**
ODI (%)				
Pre-operation	68.65 ± 12.10	70.12 ± 11.30	-0.951	0.342
Post-operation 3 days	22.34 ± 3.12	28.91 ± 3.24	-15.474	**0.001**
Final follow-up	15.56 ± 1.05	16.50 ± 1.12	-6.467	**0.001**
AVF [n (%)]	8(8.51%)	17(11.97%)	0.715	0.398
BCD [n (%)]	6(6.38%)	21(14.80%)	3.944	**0.047**

AVF, adjacent vertebral fracture; BCD, bone cement displacement. Bold values indicate *P* < 0.05, considered statistically significant.

## 4 Discussion

The present study investigated the association between the VBQ score and spongy bone cement distribution, as well as identified independent influencing factors of OVCFs patients treated with PVA. A significant association was observed between the VBQ score and spongy bone cement distribution. The VBQ score was a significant predictor (OR = 0.179, 95% CI 0.053–0.602, *P* = 0.005), with an optimal cutoff of 3.155, an AUC of 0.759 (95% CI 0.697–0.821), sensitivity of 0.723, and specificity of 0.732, where scores below 3.155 were associated with spongy cement distribution, while scores above 3.155 indicated a reduced likelihood of it. Additionally, VCR, IVC and cement volume were associated with a lower likelihood of achieving a spongy pattern. While, the BMD T-score and the PVP surgical technique were associated with a higher likelihood. Furthermore, the VBQ score was negatively correlated with the BMD T-score, suggesting that the VBQ score may reflect OP from a non-radiological perspective. During follow-ups, the spongy patients demonstrated better pain relief and functional recovery, with a lower incidence of complications.

The distribution pattern of bone cement within the surgical vertebra is a critical factor influencing postoperative outcomes. Consistent with previous studies (16–18, 30), we found that a spongy cement distribution pattern was associated with superior pain relief and functional recovery compared with a non-spongy pattern. Polymethyl methacrylate (PMMA), the primary material widely used in PVA treatment, alleviates pain through multiple mechanisms, including mechanical stabilization of the fractured vertebra, as well as chemical toxicity and thermal necrosis of adjacent sensory nerve endings (31). Compared with the non-spongy pattern, the spongy distribution pattern allows greater interdigitation of PMMA with the cancellous bone, resulting in a larger bone cement interface. This enhanced interfacial contact not only improves the mechanical stability of the augmented vertebra but may also strengthen the chemical and thermal effects on sensory nerve endings, thereby contributing to improved analgesic efficacy. A three-dimensional finite element analysis conducted by Liang et al. demonstrated that insufficient cement distribution could increase the von Mises stress of the surrounding cancellous bone, which compromises the stability of bone-cement interface (32). Under long-term mechanical loading, micro-fractures and micromotion can develop between the cement and cancellous bone, ultimately leading to BCD (28). This mechanism may explain why the incidence of BCD is lower in patients with a spongy cement distribution pattern than in those with a non-spongy pattern in the present study. This finding represents a key clinical benefit of achieving a spongy cement distribution, as BCD is a major complication that can lead to recurrent pain and the need for revision surgery. Moreover, even distribution of bone cement that contacts both the upper and lower endplates could facilitate axial stress transmission sequentially from the upper endplate to the cement and then to the lower endplate (33). This stress-sharing mechanism reduces the risk of stress fractures between cancellous bone and cement, which decrease the occurrence of BCD. Tanigawa et al. reported that non-spongy cement distribution is associated with a higher incidence of AVF during PVP for OVCFs (34). However, a spongy cement distribution pattern integrates well with the trabecular architecture of the vertebral body, balancing biological stress and reducing localized stress on adjacent endplates. This effect may contribute to the lower incidence of AVF observed in the spongy group compared with the non-spongy group in our study (35). However, the difference in AVF incidence between the two groups (8.51% vs. 11.97%) did not reach statistical significance. This may be due to the relatively small sample size and the low overall event rate. Our study may not have been adequately powered to detect a significant difference in this specific outcome. Future large-scale prospective studies with longer follow-up are needed to clarify the relationship between cement distribution and AVF risk.

Multiple factors influence the distribution of bone cement during PVA, among which overall BMD is the most critical determinant (36). Additional contributors include the presence of Schmorl’s nodes, IVC, viscosity of PMMA, and variations in injection technique (37, 38). Although dual-energy X-ray absorptiometry (DXA) has traditionally been regarded as the gold standard for assessing bone quality, lumbar degenerative changes such as vertebral osteophyte formation and bone sclerosis can lead to measurement inaccuracies and an overestimation of true BMD (39, 40). Notably, Schuit et al. reported that more than half of patients with OVCF may not be accurately identified as osteoporotic based on DXA evaluation alone (41). Furthermore, DXA entails radiation exposure and additional costs. A growing number of studies have demonstrated that the VBQ score serves as a valuable indicator of bone mass and osteoporotic fractures, with the distinct advantage of leveraging preoperative lumbar MRI without incurring extra expense or radiation (19, 27, 42, 43). Previous investigations have validated the predictive value of the VBQ score for cage subsidence after lumbar interbody fusion, pedicle screw loosening, and the occurrence of adjacent segment disease (44–48). To our knowledge, the present study is the first to explore the relationship between the VBQ score and bone cement distribution patterns following PVP. Notably, the ROC curve analysis demonstrated that the VBQ score (AUC = 0.759), VCR (AUC = 0.791), LCA (AUC = 0.798), and cement volume (AUC = 0.807) exhibited comparable predictive accuracy for identifying patients likely to achieve a spongy cement distribution, suggesting that each of these factors contributes meaningfully to the prediction model. In contrast, IVC (AUC = 0.438) and the PVP surgical technique (AUC = 0.568) showed poor predictive performance. This finding suggests that although IVC was identified as an independent risk factor in the multivariate analysis, its discriminatory ability is limited when used alone. Similarly, while PVP was associated with a higher likelihood of achieving a spongy pattern (OR = 4.914), its low AUC indicates that it should not be used as a sole predictor in clinical decision-making.

Previous VBQ studies have predominantly focused on pedicle screw loosening after spinal fusion surgery and vertebral fracture risk prediction. In contrast, our study is the first to extend the application of the VBQ score to predicting cement distribution patterns following PVA. This represents a novel translational application of the VBQ score: rather than predicting hardware-related complications or fracture risk, both of which are passive outcomes, we demonstrate that the VBQ score can serve as a preoperative decision-support tool to anticipate a modifiable surgical outcome, namely cement distribution quality. This finding bridges the gap between bone quality assessment and intraoperative cement behavior, offering a clinically actionable parameter that may help surgeons optimize surgical planning, including the choice between PVP and PKP and the adjustment of cement injection strategies. Our results demonstrated an inverse correlation between the VBQ score and BMD (r=-0.213, *P* < 0.05), consistent with prior literature (49). This modest correlation is not unexpected, as DXA and VBQ assess fundamentally different dimensions of bone quality. DXA measures bone mineral density, which can be falsely elevated by degenerative spinal changes such as osteophytes, endplate sclerosis, and facet joint hypertrophy—common findings in the elderly osteoporotic population (37, 38). In contrast, the VBQ score primarily reflects bone marrow adiposity on T1-weighted MRI, which is a marker of trabecular microstructural integrity rather than mineral content (18, 46). These two metrics therefore capture complementary but distinct aspects of bone health: one quantifies bone quantity (mineral density), while the other estimates bone quality (marrow composition and trabecular architecture). This distinction may explain why the VBQ score demonstrated independent predictive value for cement distribution patterns beyond what was captured by BMD alone in our multivariate analysis. Specifically, an elevated VBQ score, reflecting poorer vertebral bone quality, was associated with a significantly lower probability of obtaining a favorable spongy cement pattern. The underlying mechanisms can be postulated from two interrelated perspectives. First, osteoporotic bone is histologically characterized by trabecular atrophy and increased marrow adiposity (50). As vertebral porosity increases with progressive bone loss, the trabecular network which normally serves as a natural scaffold that guides cement interdigitation via capillary action, becomes sparse and discontinuous (51). Consequently, the injected cement tends to flow along the path of least resistance rather than spreading evenly along the trabecular surfaces, ultimately forming a non-spongy configuration. Conversely, higher BMD reflects denser trabecular architecture with lower porosity. In such vertebrae, the abundant and interconnected trabeculae provide extensive surface area and capillary channels that facilitate a more homogeneous, interdigitated cement distribution. Second, a higher VBQ score denotes greater marrow adipocyte infiltration. This lipid-rich environment impedes cement dispersion through a dual barrier: the hydrophobic nature of both PMMA and adipocytes generates strong hydrophobic–hydrophobic repulsion at the interface, restricting extensive cement spread and trabecular interlacing. Simultaneously, during cement injection, the displaced fatty marrow tends to encapsulate the injected cement, forming a physical isolation layer that prevents direct bone–cement contact and further inhibits a spongy distribution pattern. Taken together, these factors provide a plausible pathophysiological basis for the observed association between elevated VBQ scores and a reduced likelihood of achieving a spongy cement distribution. Future *in vitro* studies using controlled bone models are needed to test this hypothesis.

Notably, our findings also demonstrated that the PVP technique (OR = 4.914, 95% CI 1.374-17.574, *P* = 0.014) was independently associated with a significantly higher probability of achieving a spongy cement distribution compared with PKP. This may be explained by the fact that balloon inflation during PKP compresses the surrounding cancellous bone, resulting in the formation of a dense peripheral shell that restricts cement permeation beyond the created cavity (52). Patients with elevated VBQ scores, who typically exhibit poorer bone quality and a more fragile trabecular microarchitecture. This mechanical compaction effect may further limit cement interdigitation within the vertebral body. In contrast, PVP allows direct low-pressure injection of cement into the trabecular spaces, thereby facilitating a more diffuse and interdigitated cement distribution. Additionally, the viscosity of PMMA at the time of injection, which is controlled by the ‘‘wire-drawing’’ phase in our surgical protocol, may also influence cement distribution patterns. Higher viscosity cement tends to form a more compact mass with limited interdigitation, whereas lower viscosity cement may penetrate more extensively into the trabecular network. However, the optimal viscosity for achieving a spongy pattern requires further investigation, and the interplay between cement viscosity and vertebral bone quality warrants future biomechanical studies. These findings suggest that, particularly in patients with high VBQ scores, PVP may represent a more favorable surgical strategy for maximizing the likelihood of achieving a spongy cement distribution and its associated clinical benefits.

In addition to the VBQ score, four other factors including VCR (OR = 0.770, 95% CI 0.677-0.875, *P* = 0.001), LCA (OR = 0.750, 95% CI 0.667-0.884, *P* = 0.001), IVC (OR = 0.171, 95% CI 0.045-0.648, *P* = 0.009), and cement injection volume (OR = 0.247, 95% CI 0.142–0.430, *P* = 0.001), were associated with a lower likelihood of achieving a spongy pattern. Higher VCR and LCA reflected more severe vertebral collapse and kyphotic deformity, which may disrupt the intrinsic trabecular architecture and reduce residual cancellous bone connectivity (16). These structural alterations can limit PMMA interdigitation and peripheral cement dispersion, thereby promoting central cement aggregation and resulting in a non-spongy cement distribution pattern. IVC is frequently observed in patients with OVCFs and Kümmells disease (53). The fibrous tissue and sclerotic bone interface surrounding the cleft may further impede cement infiltration into adjacent cancellous bone, contributing to localized cement accumulation (16, 54). Similarly, Masaki et al. reported that non-spongy cement distribution patterns were more commonly observed in OVCFs patients with IVC (55), further supporting our results. The relationship between cement volume and cement distribution is non-linear (56). An appropriate volume sufficiently fills trabecular channels to enable interdigitation, whereas both inadequate and excessive volumes compromise dispersion. Insufficient volume fails to reach the peripheral trabecular network, leaving it unfilled, whereas excessive volume preferentially accumulates in central cavities or along low-resistance pathways, forming a non-spongy mass and increasing the risk of cement leakage. Using digital volume reconstruction, Zhou et al. demonstrated that extensive cement distribution can be achieved when the volume fraction reaches approximately 28.58% (sensitivity 72.2%, specificity 91.7%). Therefore, moderate and targeted cement injection, rather than simply increasing the total volume, is critical for achieving a spongy pattern and reducing complications such as bone cement leakage (36).

This study has several limitations. First, the optimal VBQ cut-off value of 3.155 was derived from a single-center study, which may limit the generalizability of the results and require external validation before clinical implementation. Second, the potential mechanisms underlying the association between the VBQ score and cement distribution were not directly validated by biomechanical or histological analyses. Third, although inter-observer reliability was excellent for both the VBQ score and cement distribution classification, some degree of variability remains inherent to these subjective assessments. Fourth, the calibration of the logistic regression model was assessed using the Hosmer-Lemeshow test, indicating adequate fit. However, external validation in an independent cohort is needed. Fifth, the follow-up period was relatively short, and longer-term outcomes, including late AVF incidence and functional decline, require further investigation. Sixth, the study did not include a control group of patients without OVCFs, which limits the ability to assess the diagnostic performance of the VBQ score for osteoporosis screening. Seventh, subgroup analyses were not performed due to insufficient sample size for meaningful comparisons, and these questions warrant investigation in future multicenter studies with larger cohorts. Eighth, the exclusion of patients with multi-segmental vertebral fractures may limit the generalizability of our findings to patients with multiple fractures, and future studies are needed to validate these results in such populations.

The VBQ score may serve as a useful preoperative predictor of bone cement distribution in patients with OVCFs undergoing PVA. Patients with higher VBQ scores (>3.155) were more likely to exhibit a non-spongy cement distribution pattern, which may be associated with poorer postoperative recovery. Therefore, the VBQ score could assist in preoperative risk stratification and individualized surgical planning. For patients with high VBQ scores and poor bone quality, surgeons may consider optimizing surgical techniques, including the choice of PVP and adjustment of cement injection strategies, to improve cement dispersion and clinical outcomes. Further prospective multicenter studies are needed to validate these findings.

## 5 Conclusion

The preoperative VBQ score is a simple, non-invasive, and reliable predictor of spongy cement distribution pattern following PVA in patients with OVCFs. The VBQ score, LCA, VCR, IVC, and higher cement volume were associated with a lower likelihood of achieving a spongy pattern, whereas higher BMD T-score and the PVP technique were associated with a higher likelihood. These findings may assist surgeons in preoperative risk stratification and surgical planning.

## Data Availability

The raw data supporting the conclusions of this article will be made available by the authors, without undue reservation.
